# Online Information on Electronic Cigarettes: Comparative Study of Relevant Websites From Baidu and Google Search Engines

**DOI:** 10.2196/14725

**Published:** 2020-01-24

**Authors:** Ting Chen, Sarah Gentry, Dechao Qiu, Yan Deng, Caitlin Notley, Guangwen Cheng, Fujian Song

**Affiliations:** 1 School of Public Health Hubei Provincial Key Laboratory of Occupational Hazard Identification & Control Wuhan University of Science & Technology Wuhan China; 2 Norwich Medical School University of East Anglia Norwich United Kingdom

**Keywords:** electronic nicotine delivery system, electronic cigarette, online health information, internet-based information

## Abstract

**Background:**

Online information on electronic cigarettes (e-cigarettes) may influence people’s perception and use of e-cigarettes. Websites with information on e-cigarettes in the Chinese language have not been systematically assessed.

**Objective:**

The aim of this study was to assess and compare the types and credibility of Web-based information on e-cigarettes identified from Google (in English) and Baidu (in Chinese) search engines.

**Methods:**

We used the keywords *vaping* or *e-cigarettes* to conduct a search on Google and the equivalent Chinese characters for Baidu. The first 50 unique and relevant websites from each of the two search engines were included in this analysis. The main characteristics of the websites, credibility of the websites, and claims made on the included websites were systematically assessed and compared.

**Results:**

Compared with websites on Google, more websites on Baidu were owned by manufacturers or retailers (15/50, 30% vs 33/50, 66%; *P*<.001). None of the Baidu websites, compared to 24% (12/50) of Google websites, were provided by public or health professional institutions. The Baidu websites were more likely to contain e-cigarette advertising (*P*<.001) and less likely to provide information on health education (*P*<.001). The overall credibility of the included Baidu websites was lower than that of the Google websites (*P*<.001). An age restriction warning was shown on all advertising websites from Google (15/15) but only on 10 of the 33 (30%) advertising websites from Baidu (*P*<.001). Conflicting or unclear health and social claims were common on the included websites.

**Conclusions:**

Although conflicting or unclear claims on e-cigarettes were common on websites from both Baidu and Google search engines, there was a lack of online information from public health authorities in China. Unbiased information and evidence-based recommendations on e-cigarettes should be provided by public health authorities to help the public make informed decisions regarding the use of e-cigarettes.

## Introduction

Electronic cigarettes (e-cigarettes) are also called electronic nicotine delivery systems or nicotine vaping products, although nicotine-free e-cigarettes exist. Users of e-cigarettes inhale a vapor that may contain nicotine by heating a solution with battery power. Since the first patent of e-cigarettes in 2003 in China, different types of e-cigarettes have been developed [1], and 80% of the global e-cigarette products are manufactured in China [2]. More recently, *heat-not-burn* tobacco (HNBT) products have been developed to electronically heat processed tobacco to produce aerosol without combustion [3], and they have been increasingly used in Japan and many other countries [4].

Along with dramatically increased popularity, the potential impact of e-cigarettes on public health remains controversial [5]. Available research evidence indicates that the use of e-cigarettes is considerably less harmful than the use of traditional combustible cigarettes; hence, e-cigarettes have been recommended for smokers as cessation aids or harm reduction alternatives [6]. However, some researchers or tobacco control experts remain skeptical about the impact of e-cigarettes on public health for the following reasons. First, e-cigarettes are not risk free, and there is limited evidence on the effects of e-cigarettes for smoking cessation [7]. Second, possible *gateway effects* may increase smoking initiation among young people [8,9]. E-cigarettes may be categorized differently as tobacco products, therapeutic products, or consumer products by authorities in different countries [10]. Although the sale and use of e-cigarettes are unregulated in many countries (eg, mainland China), they have been banned for sale in some countries (eg, Australia and Brazil) but encouraged for smoking cessation or harm reduction in others (eg, United Kingdom) [11,12].

There are very different prevalence rates of use of e-cigarettes across countries [5]. The prevalence of current use of e-cigarettes in 2018 was 6.3% in England [13] and only 0.9% in China [14]. The high prevalence of current use of e-cigarettes in the United Kingdom could be explained by various public health and health organizations’ recommendation of e-cigarettes as cessation aids or less harmful alternatives for smokers. Nonetheless, the very low prevalence of current use of e-cigarettes in China is noteworthy, which is contrasting to a high prevalence of cigarette smoking, largely unregulated e-cigarette marketing, and substantial amount of global e-cigarette products made in China [2].

Online advertising is an important approach for manufacturers and retailers to marketing e-cigarettes and accessories. A systematic review found an association between exposure to online marketing and intention to use or trial use of e-cigarettes [15]. Furthermore, the internet remains an important source of health information for the general public, and the increased availability of mobile phones has facilitated the internet access globally [16]. A study found that 80% of those studied used internet search engines to look for information on e-cigarettes, and online information may influence people’s perception and use of e-cigarettes [17].

A study evaluated websites of e-cigarette manufacturers in China and concluded that e-cigarette marketing should be better regulated [18]. However, online information on e-cigarettes in Chinese has not been systematically assessed. This study aimed to assess and compare types and credibility of Web-based information on e-cigarettes identified from Google (in English language) and Baidu (in Chinese language) search engines.

## Methods

### Search for and Inclusion of Relevant Websites

We found no studies on keywords used by ordinary users to search for online information on e-cigarettes. The term *electronic cigarette* was used in a study of websites of e-cigarette manufacturers in China [18]. Therefore, we used keywords *vaping* or *e-cigarettes* for Google search engine and equivalent Chinese characters for Baidu search engine. The Google search was conducted in England, and the Baidu search was conducted in Wuhan, China, on January 21, 2019. The Google search provided approximately 19 million results, and the Baidu search showed approximately 37 million results. Considering that typical internet users would focus on a limited number of Web search pages and restrictions of available time and other resources, we selected the first 50 unique and relevant websites from each of the 2 search engines, after excluding duplicates, irrelevant websites, and those that were no longer active.

### Data Extraction and Analysis

We developed a data extraction form, which was tested and revised using the first 4 included websites from each of the 2 search engines (Multimedia Appendix 1). Then, 2 authors independently extracted information from included websites (TC, YD, and GC from Baidu websites and SG and DQ from Google websites). To ensure consistency in data extraction, a third author (FS) compared and checked all extracted data and made an arbitrary decision for any disagreements between reviewers.

We extracted the following data from the relevant websites: characteristics of the website, main messages regarding the use of e-cigarettes, and website credibility (Multimedia Appendix 1). Claims regarding e-cigarettes were categorized as smoking cessation claims, health claims, social claims, and age restriction warnings, according to the method used by Hsu et al [1]. From e-cigarette advertising websites, we collected data on types of e-cigarettes, including Cigalike (closed system), eGo (open system), and Mods (advanced personal vaporizers). We also recorded whether HNBT products were mentioned, although HNBT are products different from e-cigarettes.

Quality of included websites was assessed using the Quality Evaluation Scoring Tool (QUEST) [19]. The QUEST has been validated in a previous study and can be used to assess the quality of online information from the following 6 aspects: authorship, attribution, conflict of interest, currency, complementarity, and tone of claims. We removed the *complementarity* item for patient-physician relationship from the QUEST tool because it is of limited relevance in this study. In addition, attribution (evidence source) was revised to distinguish single and multiple studies and systematic reviews. For a website, the quality score ranges from 0 to 28, in which a higher score indicates a better website quality (see Multimedia Appendix 1 for more details).

We summarized data extracted from included websites in tables and reported percentages of the main website characteristics. Differences in the proportion of websites with certain characteristics between Google and Baidu search engines were tested using Pearson chi-square test or Fisher exact test if any of the expected cell sizes were less than 5. Two-sample Wilcoxon rank-sum method was used to test the difference in the total QUEST score between websites from Google and Baidu search engines. Statistical significance was defined as 2-sided *P*≤.05. Stata/Special Edition 14.2 (StataCorp LLC, USA) software was used for statistical analysis.

## Results

### Characteristics of Included Websites

The main characteristics of included websites are shown in Table 1. All the included websites from Baidu search engine were located in mainland China. For the websites from Google search engine, 24 were located in the United States, 20 in the United Kingdom, and 6 in other countries (including 1 each in Australia and Canada, 2 in New Zealand, and 2 with unclear locations). Providers of the included websites from Baidu search engine were mostly e-cigarette manufacturers/retailers (33/50, 66%) and mass media or information technology (IT) services (15/50, 30%). None of the included websites from Baidu search engine were owned by public or health professional institutions. For the 50 websites from Google search engine, 12 (24%) were owned by public or health professional institutions, 15 (30%) by e-cigarette manufacturers or retailers, 12 (24%) by media or IT companies, and 11 (22%) by charity or not-for-profit nongovernment organizations (Table 1).

Compared with websites from Google search engine, those from Baidu search engine were more likely to contain e-cigarette advertising (37/50, 74% vs 15/50, 30%; *P<*.001) and user feedback (21/50, 42% vs 11/50, 22%; *P*=.03), but they were much less likely to provide information on health education (3/50, 6% vs 30/50, 60%; *P*<.001). In addition, relatively more Baidu websites provided e-cigarette–related news, although the difference in the proportion was statistically nonsignificant (*P*=.14). Contents covered by the included websites were similar from the 2 search engines, including descriptions about what are e-cigarettes, e-cigarettes’ harm or risk, and the role of e-cigarettes for smoking cessation (Table 1). However, websites from Baidu search engine were less likely to consider regulation issues than those from Google search engine (*P*=.04).

**Table 1:** The main characteristics of included websites.

Characteristics		Baidu (N=50), n (%)	Google (N=50), n (%)	*P* value
**Country**				—^a^
	China	50 (100)	0 (0)	
	United States	0 (0)	24 (48)	
	United Kingdom	0 (0)	20 (40)	
	Other	0 (0)	6 (12)	
**Website owner^b^**				<.001
	Public or health professional institutions	0 (0)	12 (24)	
	Manufacturer/retailer	33 (66)	15 (30)	
	Media/information technology services	15 (30)	12 (24)	
	Charity/not-for-profit nongovernment organization/other	2 (4)	11 (22)	
**Type of information^c^**				
	Health education	3 (6)	30 (60)	<.001
	News	21 (42)	14 (28)	.14
	Advertisement	37 (74)	15 (30)	<.001
	Blogs/user feedback	21 (42)	11 (22)	.03
**Content coverage^c^**				
	What are e-cigarettes^d^	31 (62)	35 (70)	.40
	E-cigarettes’ harm	32 (64)	40 (80)	.08
	E-cigarettes for quitting	35 (70)	31 (62)	.40
	E-cigarette regulation	23 (46)	33 (66)	.04

^a^Not applicable.

^b^*P* value for the website owner was a test of null hypothesis that there was no statistically significant difference in the proportion of website owners between Baidu and Google search engines.

^c^As individual items were not mutually exclusive for type of information and content coverage, *P* values for items belonging to these 2 variables were tests of null hypothesis that there was no statistically significant difference in the proportion of an individual item between the 2 search engines.

^d^e-cigarettes: electronic cigarettes.

### Quality of Included Websites

The included websites from Baidu search engine had lower modified QUEST scores than those from Google search engine. The median QUEST score was 5 (range: 0-16) for the Baidu websites and 15 (range: 0-27) for the Google websites (*P*<.001). After excluding websites owned by manufacturers or retailers, the median QUEST score was 6.3 (range: 2-11) for the Baidu websites and 18.8 (range: 9-27) for the Google websites (*P*<.001). Baidu websites tended to be more current (ie, more recently updated) than Google websites (Table 2). However, compared with Google websites, Baidu websites were associated with a higher proportion of no indication of authorship, lacking or inadequate scientific evidence, high risk of conflict of interest, and fully supportive tone of claims (Table 2). For a subgroup analysis of websites not owned by manufacturers or retailers, the differences in quality between Baidu and Google websites were increased for attribution, high risk of conflict of interest, and tone of claims (Table 2). It is noteworthy that information was considered to be unbiased for 20 of the 35 Google websites that were not owned by manufacturers or retailers, whereas information was of high or unclear risk of bias for all of the Baidu websites (Table 2).

**Table 2:** Modified Quality Evaluation Scoring Tool website quality criteria and scores. For categorical variables with items that were mutually exclusive, a single *P* value was obtained from chi-square test. *P* values for the Quality Evaluation Scoring Tool score were based on Wilcoxon rank-sum test.

Criteria		All included websites			Nonmanufacturers or nonretailers		
		Baidu (N=50), n (%)	Google (N=50), n (%)	*P* value	Baidu (N=17), n (%)	Google (N=35), n (%)	*P* value
**Authorship**				.008			.13
	No indication of authorship	44 (88)	33 (66)		12 (71)	18 (51)	
	All other indications of authorship	6 (12)	10 (20)		5 (29)	10 (29)	
	Author/qualification clearly stated	0 (0)	7 (14)		0 (0%)	7 (20)	
**Attribution—a**				<.001			<.001
	No sources	28 (56)	9 (18)		10 (59)	0 (0)	
	Mention of expert source and research findings, but insufficiently	20 (40)	14 (28)		7 (41)	12 (34)	
	Reference to at least one identifiable scientific study	2 (4)	10 (20)		0 (0)	6 (17)	
	Reference to mainly identifiable scientific studies	0 (0)	17 (34)		0 (0)	17 (49)	
**Attribution—b**				<.001			.001
	Not available; in vitro, animal, and editorials	46 (92)	23 (46)		15 (88)	14 (40)	
	Single journal article	3 (6)	2 (4)		2 (12)	1 (3)	
	Multiple journal articles	1 (2)	15 (30)		0 (0)	13 (37)	
	Systematic reviews of studies	0 (0)	10 (20)		0 (0)	7 (20)	
**Conflicts of interest**				<.001			<.001
	High risk of conflict of interest	27 (54)	15 (30)		5 (29)	1 (3)	
	Unclear risk of conflict of interest	23 (46)	15 (30)		12 (71)	14 (40)	
	Unbiased information	0 (0)	20 (40)		0 (0)	20 (57)	
**Currency**				.001			.006
	No date present	7 (14)	25 (50)		0 (0)	12 (34)	
	Dated but 1 year old or older	7 (14)	3 (6)		0 (0)	3 (9)	
	Dated within the last 1 year	36 (72)	22 (44)		17 (100)	20 (57)	
**Tone of claims**				<.001			<.001
	Fully supported	36 (72)	12 (24)		14 (82)	3 (9)	
	Mainly supported	13 (26)	17 (34)		3 (18)	12 (34)	
	Balanced/cautious support	1 (2)	21 (42)		0 (0)	20 (57)	

### Claims on the Included Websites

Table 3 shows claims or messages on the included websites, according to whether websites were owned by manufacturers or retailers. For websites owned by manufacturers/retailers, Baidu websites were more likely to claim that the use of e-cigarettes helped with smoking cessation (22/33, 67% vs 5/15, 33%) and that e-cigarettes were less harmful than combustible cigarettes (20/33, 61% vs 7/15, 47%), although the differences between the 2 search engines were statistically nonsignificant (*P*>.05). Google websites owned by manufacturers/retailers were more likely to claim that the use of e-cigarettes was cheaper than the use of combustible cigarettes (7/15, 47% vs 3/33, 9%; *P*=.006). An age restriction warning was shown on all Google websites owned by manufacturers or retailers but by only on 10 of the 33 (30%) Baidu websites owned by manufacturers or retailers (*P*<.001).

After excluding websites owned by manufacturers or retailers, the differences in quitting, health claims, and social claims between Baidu and Google websites were statistically nonsignificant (Table 3). However, 3 of the 17 Baidu websites that were not owned by manufacturers or retailers claimed that e-cigarettes were more harmful than combustible cigarettes, compared with only 1 of the 35 such Google websites (*P*=.21).

**Table 3:** Claims or messages from the included websites.

Claims		Manufacturer/retailer websites			Nonmanufacturer or nonretailers			
		Baidu (N=33), n (%)	Google (N=15), n (%)	*P* value		Baidu (N=17), n (%)	Google (N=35), n (%)	*P* value
**Electronic cigarette for quitting^a^**								
	Help quit	22 (67)	5 (33)	.053		8 (47)	14 (40)	.86
	Not help quit	1 (3)	0 (0)	—^b^		2 (12)	7 (20)	—
	Unclear/other	10 (30)	10 (67)	—		7 (41)	14 (40)	—
**Health claims^a^**								
	Healthier than cigarettes	20 (61)	7 (47)	.53		7 (41)	16 (46)	.21
	More harmful than cigarettes	0 (0)	0 (0)	—		3 (18)	1 (3)	—
	Unclear/other	13 (39)	8 (53)	—		7 (41)	18 (51)	—
**Social claims (multiple choices allowed)^c^**								
	Less expensive than cigarettes	3 (9)	7 (47)	.006		0 (0)	2 (6)	>.99
	Cleaner than cigarettes	14 (42)	4 (27)	.35		4 (24)	4 (11)	.41
	More socially acceptable	5 (15)	3 (20)	.69		1 (6)	6 (17)	.40
**Age claims/warning ^a^**								
	Yes	10 (30)	15 (100)	<.001		2 (12)	29 (83)	<.001
	No	23 (70)	0 (0)	—		15 (88)	6 (17)	—

^a^When items belonging to a variable were mutually exclusive, a single *P* value was obtained from chi-square test for the variable (ie, e-cigarette for quitting, health claims, and age claims).

^b^Not applicable.

^c^If items for a variable were not mutually exclusive (ie, social claims), a *P* value was shown for each row from a test of null hypothesis that there was no statistically significant difference in the proportion of the item between the 2 search engines.

### Types of Electronic Cigarettes Advertised

Table 4 shows types of e-cigarettes advertised on websites from Baidu and Google search engines. Relatively more Google websites were advertising first-generation e-cigarettes than Baidu websites, although the difference was statistically nonsignificant (*P*=.07). The second- and third-generation types of e-cigarettes were similarly promoted on most advertising websites from both Baidu and Google search engines. However, HNBT products were advertised in much more Baidu websites than Google websites (24/37, 65% vs 1/15, 7%; *P*<.001).

**Table 4:** Types of electronic cigarettes on advertising websites.

Types of electronic cigarettes	Baidu (N=37), n (%)	Google (N=15), n (%)	*P* value
Cigalike	14 (38)	10 (67)	.07
Advanced personal vaporizers or eGo	27 (73)	14 (93)	.15
Mods	30 (81)	13 (87)	.63
*Heat-not-burn* tobacco	24 (65)	1 (7)	<.001

## Discussion

### Prinicpal Findings

Findings of this study revealed that the overall credibility and quality of the included Baidu websites were much lower than those of the included Google websites. Compared with Google websites, the included Baidu websites were more likely to be owned by manufacturers or retailers, more likely to advertise HNBT products, less likely to focus on health education, and less likely to have an age restriction warning. The included websites from the 2 search engines similarly provided conflicting or unclear claims regarding whether the use of e-cigarettes helped smoking cessation and whether the use of e-cigarettes was more or less harmful and more or less socially acceptable compared with the use of conventional cigarettes.

Claims made on the marketing websites included in this study were similar to those identified by previous studies, including claims that e-cigarettes could help smoking cessation and that e-cigarettes are safer, cheaper, cleaner, and more socially acceptable than combustible cigarettes [15,18,20,21]. However, conflicting or unclear health and social claims about e-cigarettes were made on nonmanufacturer/retailer websites from both Baidu and Google search engines. The public may trust online information from public health authorities more than the online information from manufacturers and retailers. None of public health authority websites were identified from Baidu search engine. From Google search engine, 3 public health authorities’ websites (1 each from Canada [22], the United States [23], and the United Kingdom [24]) clearly support the use of e-cigarettes for smoking cessation and indicate that the use of e-cigarettes was less harmful than cigarette smoking. According to information on 1 of the included Baidu websites (Baidu Wikipedia website on e-cigarettes, endorsed by Chinese Society of Preventive Medicine [25]), the effect of e-cigarettes for smoking cessation was uncertain and the use of e-cigarettes was more harmful than cigarette smoking. Although many advertising websites from Baidu search engine had positive claims on the use of e-cigarettes, there were no credible websites in Chinese language that supported the use of e-cigarettes for smoking cessation or as a safer alternative to cigarette smoking. Further research is required to investigate whether this is a reason for a very low uptake of e-cigarettes at 0.9% in 2018 in China [14], compared with 6.3% in the United Kingdom [13].

To avoid *gateway effects* of e-cigarette use among young people, some tobacco control experts and public health agencies may stress potential harms of e-cigarettes, and they have been reluctant to support the use of e-cigarettes for smoking cessation or harm reduction [9]. Due to a lack of unbiased information from credible sources, young people in China may be more exposed to marketing information from manufacturers and retailers. E-cigarettes are often displayed as fashion or health products with modern stylish designs to attract young users [26]. Although government agencies in China ban selling of e-cigarettes to underaged young people [27], a large proportion of marketing websites from Baidu search engine had no age restriction warnings.

A public health challenge in China is the very high prevalence of cigarette smoking among men (52.1% in 2015 and 50.5% in 2018) [14]. In addition to other tobacco control measures, it is important to investigate whether the use of e-cigarettes could increase smoking cessation in China, for example, as has being observed in the United Kingdom [6]. However, further research is required to comprehensively understand the public health impact of e-cigarettes on smoking initiation in young people, cessation among current smokers, and changes in all related diseases. Currently, there is an urgent need for unbiased information and evidence-based recommendations on e-cigarettes from public health authorities in China.

### Strengths and Limitations

This is the first study to compare information on e-cigarettes between Baidu (in Chinese language) and Google (in English language) search engines. The credibility of and claims on the included websites were systematically assessed. Although the existing study had focused on advertising or marketing websites [18], this study included various websites with information on e-cigarettes.

We included only the first 50 websites from each of the 2 search engines, assuming that ordinary internet users tend to look at the first few search pages. This study considered only websites in Chinese and English language. We used simple terms (*e-cigarette* or *vaping*) to identify relevant websites, although internet users might use or add other search terms (eg, harm, risk, and quitting). Therefore, this study may have missed some influential websites.

### Conclusions

The overall quality of the included Baidu websites was much lower than that of the included Google websites, although conflicting or unclear claims on e-cigarettes were common on websites from both Baidu and Google search engines. Compared with websites from Google search engine, relatively more websites from Baidu search engine were e-cigarette advertising. Particularly, there was no credible information on e-cigarettes from public health authorities in China. Unbiased information and evidence-based recommendations on e-cigarettes should be provided by public health authorities to help the public make informed decisions regarding the use of e-cigarettes.

## Appendix

Multimedia Appendix 1 Data extraction form: assessment of Web-based information on electronic cigarettes.

## Abbreviations

e-cigarette: electronic cigarette
HNBT: heat-not-burn tobacco
IT: information technology
QUEST: Quality Evaluation Scoring Tool
