# Supplementary material for: Online Information on Electronic Cigarettes: Comparative Study of Relevant Websites From Baidu and Google Search Engines
Source: J Med Internet Res. 2020 Jan 24;22(1):e14725. doi: 10.2196/14725 (PMC7007591; doi:10.2196/14725)
Supplement: Multimedia Appendix 1 [file jmir_v22i1e14725_app1.pdf]

Supplemental Appendix 1: Data extraction form – assessment of web-based information on e-cigarettes

**Who extract data?**	Name: ________ _____
**Search engine, web site # :**	□ Google, No.:_____ □ Baidu, No: _____
**Access date:**<br>**Web site link:**	Access date:_________________<br>http://
**Who runs the Web site?**	□ Government/public/university<br>□ Health institution<br>□ E-cig industry/commercial<br>□ Information services company<br>□ Charity/not-for-profit NGO<br>□ Other: ______________________<br>□ Unclear
**Organization or company name:**	
**What types of information?**<br>(Multiple choices allowed)	□ Health education<br>□ News<br>□ Advertisement<br>□ Consumer feedback<br>□ Other: ______________________<br>□ Unclear
**Content coverage?**	What are e-cigs: □ Yes; □ No; □Unclear<br>E-cigs' harms: □ Yes; □ No; □Unclear<br>E-cigs for quitting: □ Yes; □ No; □Unclear<br>E-cig regulation: □ Yes; □ No; □Unclear<br>Other: ___________________
**For advertisement websites:**<br>**Types of e-cigarettes:**	□ Cigalike –closed system<br>□ eGo - open system<br>□ Mod –APVs<br>□ Heat not burn product<br>□ Unclear/other: ____________
**Main messages and claims from the website:**	
**Smoking cessation claims:**	□ Help quit<br>□ Not help quit<br>□ Unclear/other: ________
**Health claims:**	□ Healthier than tobacco cigarettes<br>□ More harmful than tobacco cigarettes<br>□ Unclear/other: ________
**Social claims:**<br>(Multiple choices allowed)	□ Less expensive than tobacco cigarettes<br>□ Cleaner than tobacco cigarettes<br>□ More socially acceptable than tobacco cigarettes<br>□ Unclear/other: ________
**Age claims:**	□ Not for children/young people<br>□ No age claims<br>□ Unclear/other: ________________
**Any other notes/commentary:**	
**Modified QUEST criteria/scores (range 0-28)** Reference for QUEST tool: Robillard JM, Jun JH, Lai JA, Feng TL. The QUEST for quality online health information: validation of a short quantitative tool. BMC medical informatics and decision making 2018; 18(1): 87.	

**Authorship:**<br>**(Score x1):_________**	0- No indication of authorship or username<br>1- All other indications of authorship<br>2- Author's name and qualification clearly stated
**Attribution (a):**<br>**(Score x3):_______**	0- No sources<br>1- Mention of expert source, research findings (though with insufficient information to identify the specific studies), links to various sites, advocacy body, or other<br>2- Reference to at least one identifiable scientific study, regardless of format (e.g., information in text, reference list)<br>3- Reference to mainly identifiable scientific studies, regardless of format (in >50% of claims)
**Attribution (b):**<br>**(Score x1):________**	For all articles scoring 2 or 3 on Attribution (a): Type of study<br>0- Not applicable; or in vitro, animal models, editorials<br>1- Single journal article (year: ________)<br>2- Multiple journal articles (most recent year: _________)<br>3- Systematic reviews of research studies (year:___________)
**Conflict of interest:**<br>**(Score x 3):________**	0- High risk of conflict of interest<br>1- Unclear<br>2- Unbiased information
**Currency:**<br>**(Score x 1):_________**	Web site update date (month/year): ________________________<br>0- No date present<br>1- Article is dated but 1 years or older<br>2- Article is dated within the last 1 years
**Tone (includes title):**<br>**(Score x 3):_________**	0- Fully supported (authors fully and unequivocally support the claims, strong vocabulary, no discussion of limitations)<br>1- Mainly supported (authors mainly support their claims but with more cautious vocabulary such as "can reduce your risk" or "may help prevent", no discussion of limitations)<br>2- Balanced/cautious support (authors' claims are balanced by caution, includes statements of limitations and/or contrasting findings)
**Total score:___________**	Any notes/Commentary:
